# A polyclonal allelic expression assay for detecting regulatory effects of transcript variants

**DOI:** 10.1186/s13073-020-00777-8

**Published:** 2020-09-11

**Authors:** Margot Brandt, Alper Gokden, Marcello Ziosi, Tuuli Lappalainen

**Affiliations:** 1 New York Genome Center, New York, NY USA; 2 Department of Systems Biology, Columbia University, New York, NY USA

**Keywords:** CRISPR/Cas9 genome editing, Variant validation, Regulatory variation, eQTL, Nonsense-mediated decay

## Abstract

We present an assay to experimentally test the regulatory effects of genetic variants within transcripts using CRISPR/Cas9 followed by targeted sequencing. We applied the assay to 32 premature stop-gained variants across the genome and in two Mendelian disease genes, 33 putative causal variants of eQTLs, and 62 control variants in HEK293T cells, replicating a subset of variants in HeLa cells. We detected significant effects in the expected direction (in 60% of variants), demonstrating the ability of the assay to capture regulatory effects of eQTL variants and nonsense-mediated decay triggered by premature stop-gained variants. The results suggest a utility for validating transcript-level effects of genetic variants.

## Background

The interpretation of the functional effects of common and rare variants in the human population is a major objective in human genetics and genomics. Despite the success of mapping genetic associations to complex traits by genome-wide association studies (GWAS) and interpreting their effects on gene expression by expression quantitative trait loci (eQTL) studies [1–5], the causal variants at GWAS loci and eQTLs are usually unknown due to linkage disequilibrium (LD). Statistical fine-mapping methods [6–8] can help narrow down causal variants, but experimental validation of the performance of these methods is lacking. For rare variants, functional interpretation has distinct challenges even in the well-annotated coding regions. Rare disease studies often result in hundreds to thousands of potential disease-causing variants identified from whole-exome sequencing, and prioritization based on their functional effect is essential for research and clinical use [9].

Thus, there is a need for experimental methods to confirm the effects of common and rare variants. Methods such as massively parallel reporter assays (MPRAs) [10, 11], which couple regulatory sequences with an expression-correlated reporter, are high-throughput approaches for finding active regulatory variants outside of the gene body, and analogous methods exist for variants affecting splicing [12, 13]. However, the results of the assays show low concordance with eQTL data [10, 11], perhaps due to taking the variant out of its genomic context. Furthermore, MPRAs are not suited to testing variants within the transcript that can affect gene expression levels via post-transcriptional mechanisms, e.g., RNA stability. Regulatory variants are strongly enriched not only in promoters and enhancers, but also in UTRs and other transcript annotations [1], emphasizing the need for a method to validate them. Similar mechanisms also apply to a subset of rare disease variants, where stop-gained and frameshift variants can affect transcript abundance via nonsense-mediated decay (NMD) [14]. Stop-gained variants located 50–55 bp or more before the last exon junction often induce NMD, while variants located beyond this threshold are more likely to escape NMD and therefore produce truncated protein [15]. However, these predictions are not perfect [16]. Variants that trigger or escape NMD in the same gene can manifest in diseases with different symptoms or methods of inheritance [17], making it important to validate whether a given variant induces NMD.

CRISPR/Cas9 genome editing technology [18–20] has provided a means to introduce specific variants into the genome in order to validate their effects on expression in the native genomic context. However, editing one variant at a time, isolating hundreds of single-cell clones, genotyping and expanding clones, and measuring transcript abundance are hugely time-consuming and expensive processes. In addition to the resource cost of completing such an experiment, undetected large on-target mutations [21], off-target mutations, and other clone-specific abnormalities can create noise which requires many replicates of each desired genotype. A less labor-intensive genome editing approach analyzes allelic expression in the polyclonal edited cell population and has been used to validate the effects of specific rare variants [22] and all possible mutations in a particular exon using saturation mutagenesis [23].

In this study, we decided to develop and apply a similar polyclonal approach for medium-throughput testing of the expression level effects of eQTLs in transcribed regions and rare premature stop variants. We first tested rare premature stop variants with signs of NMD in the Genotype-Tissue Expression (GTEx) v8 data to validate the ability of our assay to detect effects on transcript abundance, and then applied the assay to fine-mapped eQTLs from GTEx. Finally, we assayed premature stop variants in two Mendelian disease genes, *GLI3* and *ROR2*, to evaluate our ability to test NMD in a clinically useful context. Stop-gained variants towards the beginning of *GLI3* are associated with Greig cephalopolysyndactyly, while variants towards the end of the gene are associated with the clinically distinct Pallister-Hall syndrome [24, 25]. Stop-gained variants towards the beginning of *ROR2* are associated with the autosomal recessive Robinow syndrome, while variants towards the end of the gene are associated with autosomal dominant Brachydactyly type B1 [26, 27]. It has been hypothesized that in both genes, the disease manifestation is impacted by whether or not the variant triggers NMD. In such situations, experimental testing of NMD can be valuable for disease diagnosis and prognosis.

## Methods

### fgwas enrichment

First, we sought to establish the relevance of testing eQTL effects driven by variants within transcripts by analyzing the extent of *cis*-eQTL enrichment in functional elements of the genome. We used GTEx v6 fibroblast eQTL data and a diverse set of annotations: Gene annotations were obtained from GENCODE [28], and regulatory annotations (CTCF-binding site, enhancer, open chromatin region, promoter, promoter-flanking region, and TF binding site) were obtained from the Ensembl regulatory build release 80 [29]. Additional annotations include CADD variant consequence scores [30], SPIDEX machine learning-based prediction of splicing effects [31], experimentally validated miRNA-binding sites from Tarbase [32], 3′ UTR regulatory elements [33], and RNA-binding protein sites from CLIPdb [34]. Significant fibroblast eQTLs were analyzed for enrichment in these functional annotations using fgwas [35], with each annotation tested separately.

### Assay design

**Fig. 1:**
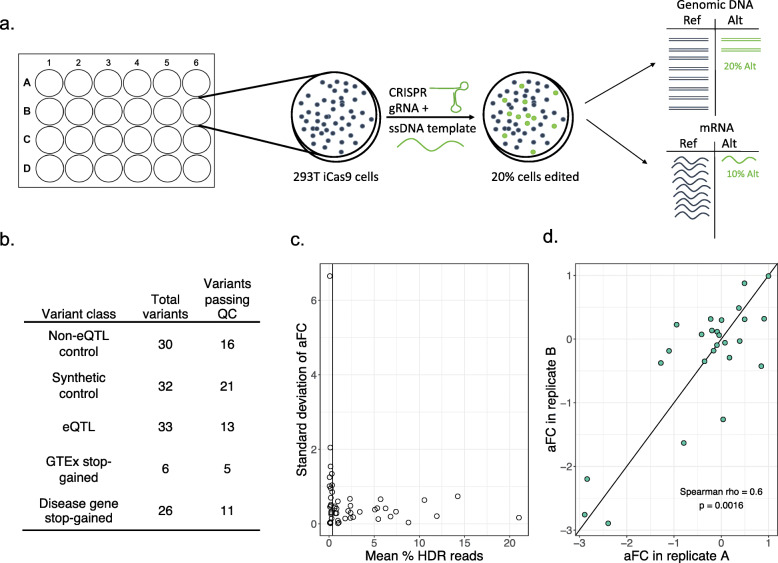
Polyclonal allelic expression assay to detect the effects of regulatory variants. **a** Assay schematic. Inducible Cas9 HEK293T cells undergo homologous recombination after transfection with the gRNA and ssDNA template in order to introduce the alternative allele to the cells. HeLa cells without inducible Cas9 were transfected with a Cas9 plasmid. Editing is followed by targeted sequencing of gDNA and mRNA to detect the ratio of alt/ref alleles in the polyclonal population of cells. **b** Table with the number of each type of control and putative regulatory variant edited with the assay in HEK293T cells. **c** Homologous recombination rate versus standard deviation for variants replicated 2–3 times with assay in HEK293T cells. The vertical line shows the 0.4% HDR cutoff which was used to filter variants for subsequent analysis. **d** Scatter plot showing the reproducibility of the effect size detected by the polyclonal allelic expression assay for two replicate experiments editing the same variants in HEK293T cells

The design of the assay is illustrated in Fig. 1a. In order to validate transcript regulatory variants’ allelic effects on transcript abundance, we utilized CRISPR/Cas9 genome editing with a gRNA specific to the locus of the variant of interest and a single-stranded DNA (ssDNA) template containing the alternative allele for homology-directed repair (HDR). For each variant of interest, we transfected the gRNA and ssDNA template into a well of inducible Cas9 human embryonic kidney cells (HEK293T). After editing, cells were harvested for gDNA and mRNA, followed by amplicon sequencing of the locus of interest in each. A regulatory effect of the variant is detected as a difference in the ratio of the alternative allele between gDNA and mRNA. This effect size is calculated as the log ratio of the alternative allele in cDNA over the ratio of the alternative allele in gDNA: log2(cDNA alt/ref/gDNA alt/ref), or the allelic fold change (aFC). Even though many cell lines are not diploid at all loci, this assay is detecting allelic events in *cis* where each homologous chromosome functions independently, and thus, variant effects are expected to be robust regardless of ploidy.

### Variant selection

In this study, we edited five types of variants: GTEx stop-gained, GTEx eQTL, disease gene stop-gained, non-eQTL synonymous control, and synthetic control variants (Fig. 1b and Additional file 1: Table S1).

Stop-gained variants from the general population were obtained from the GTEx v6 data release. Starting with all stop-gained variants that were singletons in GTEx v6, we used allele-specific expression (ASE) data from the fibroblast sample of the individual carrying the variant to select those that are likely triggering NMD. The selected variants have RNA-seq coverage of ≥ 20 reads, a reference ratio Ref/(Ref + Alt) > 0.7, and are located in a gene with > 0.5 RPKM in a published HEK293 RNA-seq dataset [36]. In HEK293 cells, 12,451 genes (61%) are expressed at the level of 0.5 > RPKM. Additionally, we required ASE data in at least 5 tissues and a first quartile of ASE across tissues of > 0.7 to select variants where NMD does not appear to be highly tissue-specific. Finally, we selected variants > 30 bp from the end of an exon for primer design. Six variants were used for editing.

eQTL variants were obtained from the GTEx v8 data release. Significant eQTL variants in fibroblasts were filtered for being within at least one protein-coding transcript, having a CAVIAR fine-mapping posterior probability of association > 0.8, an eGene with > 1 RPKM in HEK293 cells, and an effect size in the top quartile of the effect sizes of all associations (aFC > 0.30). The top 33 highest effect size variants with successful gRNA and primer design were chosen for editing.

Ten stop-gained variants for each of the disease genes *GLI3* and *ROR2* were created by changing a codon in the transcript to a stop codon. The stop codons were spaced 20 bp apart in both directions from the NMD cutoff point (55 bp upstream of last the exon-exon junction). The 6 disease variants tested were obtained from ClinVar [37], choosing disease-associated variants in the two genes on either side of the NMD threshold.

We selected 30 non-eQTL negative control variants from common synonymous variants in GTEx v8 data with an eQTL association *p* > 0.1 with the gene in which they reside. The templates for the 32 synthetic control variants were designed by introducing a nucleotide other than the reference or alternative allele at the stop-gained variant locus, which does not create a premature stop codon.

### Cell culture

Genome editing was carried out in a doxycycline-inducible Cas9 HEK293T cell line, transduced with pCW-Cas9 plasmid (Addgene plasmid #50661 [38]), courtesy of the Sagi Shapira Lab. HEK293T cells were cultured in OptiMEM (Gibco) supplemented with 5% HyClone Cosmic Calf Serum (Fisher), 1% GlutaMAX (Gibco), 1% NaPyr (Corning), and 1% penicillin/streptomycin (Corning). The cells were passaged and maintained following the standard techniques in 5% CO_2_ and 95% air.

For replication in HeLa cells, cells were cultured in DMEM (Corning) with 4.5 g/L glucose supplemented with 10% heat-inactivated FBS (Gibco), 1% GlutaMAX (Gibco), 1% NaPyr (Corning), and 1% penicillin/streptomycin (Corning).

### Genome editing

The protocol for the polyclonal editing assay can be found at 10.17504/protocols.io.7c6hize. gRNAs were designed with E-CRISP version 5.3 [39] using medium settings, with an NGG PAM, a 5′ G; excluding designs with more than 5 off-targets; and classifying off-targets as having up to 3 mismatches in the 5′ region of the gRNA. gRNAs were ordered as gBlocks gene fragments (IDT): a U6 promoter sequence followed by the specific gRNA and tracr sequence [40]. The gBlocks were amplified using the Q5 High-Fidelity 2X Master Mix (NEB) and gBlock amplification primers [40]. Homologous templates were designed by extracting the sequence 50 bp upstream and downstream of each variant and substituting the reference allele with the alternative allele. Stop-gained control templates have another nucleotide substituted in the variant position which does not create a stop codon. Homologous templates were synthesized as ultramers by IDT. If possible, primers which amplify both cDNA and gDNA were designed using the IDT primer quest, choosing those that cover the PCR target (region spanning the variant and DSB) with at least 15 bp between the PCR target and one primer and at least 60 bp to the other primer. Otherwise, cDNA- and gDNA-specific primers were designed using either the cDNA or gDNA sequence as the template. Nextera adapter sequences were appended to forward and reverse primer sequences as follows:

GTCTCGTGGGCTCGGAGATGTGTATAAGAGACAG+ForwardPrimerSequence

TCGTCGGCAGCGTCAGATGTGTATAAGAGACAG+ReversePrimerSequence

Primers were ordered as standard oligos from IDT.

Twenty-four hours before transfection for CRISPR editing, iCas9 HEK293T cells were plated in 24-well plates and induced with 5 μg/mL of doxycycline, with a separate well for each targeted variant. Cells were transfected with 500 ng homologous template and 500 ng gRNA gblock using the Lipofectamine MessengerMAX transfection reagent. After 24 h, the transfection reagent was removed and replaced with new media. Cells were split after 4 days and 6 days, and DNA and RNA were extracted from the polyclonal edited cultures at 9 days. Seventy-five percent of the 24-well culture was harvested for RNA using IBI Isolate DNA/RNA Reagent according to the manufacturer’s instructions. Purified RNA was quantified by Nanodrop (Thermo Fisher). cDNA was synthesized with ~ 200 ng of purified RNA using 1/4 reactions of SuperScript IV VILO Master Mix with EZ DNase (Invitrogen). Another 10% of the cell culture was used for DNA extraction using 15 μL of QuickExtract (Lucigen). For the timecourse optimization experiment, mRNA and gDNA were extracted as above at days 4, 6, and 9.

For editing selected variants (Additional file 2: Table S1) in HeLa cells, to confer puromycin and Cas9 expression, we used transient transfection with pCC_01 plasmid (Addgene #139086) [41]. Cells were plated in 24-well plates and transfected at 90% confluency with 500–750 ng homologous template, 500 ng gRNA gblock, and 400 ng pCC_01 vector using Lipofectamine 3000 transfection reagent. After 24 h, transfection reagent was removed and replaced with fresh medium plus puromycin (1,5μg/mL). Cells were kept under puromycin selection for 72 h, until non-transfected control cells were completely eliminated. The medium was replaced at days 4 and 6. mRNA and gDNA were extracted from the edited cultures at 9 days as described above.

### Library preparation

Amplicon libraries from cDNA and gDNA were created using either the same Nextera primers (if possible) or separate Nextera primers for cDNA and gDNA. One microliter of cDNA or gDNA was amplified using Q5 High-Fidelity 2X Master Mix (NEB). An indexing PCR was performed next using Nextera XT index kit primers (Illumina) and NEBNext High-Fidelity 2X PCR Master Mix (NEB) which resulted in dual barcoded amplicons with Illumina adapters. cDNA and gDNA libraries were mixed in equal volume and sequenced on the MiSeq using 150-bp paired-end reads. We obtained a median coverage of about 85,000 reads per sample.

### Sequencing analysis

Fastqs generated from Illumina software were trimmed for adapter sequences and quality using trimmomatic. Reads were aligned to the gDNA or cDNA sequence specific for each amplicon and categorized as HDR, no edit, or NHEJ using EdiTyper [42]. Variants were eliminated if HDR in gDNA was greater than 30% (suggesting the cell line is in fact heterozygous for the variant). Samples were filtered out if they had fewer than 1000 reads covering the locus of interest. Additionally, samples were filtered out if they had an outlier NHEJ rate of greater than 80%, indicative of an alignment error. Reference and alternative allele counts were obtained from the EdiTyper results (no edit and HDR reads, respectively) for each cDNA and gDNA sample (Additional file 2: Table S2). The effect size for each variant was calculated as the log_2_((Alt/Ref in cDNA)/(Alt/Ref in gDNA)), or allelic fold change (aFC). An effect size of zero means the variant has no effect on transcript abundance.

### Statistical analysis

Significance between the control variant distribution and the other experimental variant types was determined using a two-sided Wilcoxon rank sum test. An *F* test was utilized to detect a difference in the variance of aFC between non-eQTL control and synthetic control variants, and eQTL and control variants. For each individual regulatory variant, a *p* value was calculated from the *z*-score of the variant’s effect size based on the mean and standard deviation of the control distribution. The *p* values were then Bonferroni corrected, and variants with a corrected *p* value of less than 0.05 were considered significant.

### eQTL effect size in GTEx

For the GTEx effect sizes for the eQTLs, we used the allelic fold change (aFC) estimates from the GTEx v8 data release [4, 43, 44]. For the eQTL effect size in each GTEx tissue, we used the aFC estimates calculated from eQTL data. For stop-gained variants, we calculated aFC as the log_2_ ratio of the alternative and reference allele counts in the RNA-seq data in GTEx. To analyze the variation of eQTL variants’ effects across GTEx individuals, we calculated the aFC for each eQTL variant across heterozygous individuals in GTEx as the ratio of allele counts in the gene body [43]. Samples were filtered for those with greater than 50 reads covering heterozygous sites in the gene.

## Results

First, we assessed the right time point to harvest mRNA after transfection with CRISPR constructs. Since mRNA is likely to remain in the cell for hours to days after editing has occurred, we expect to see fewer edited mRNA molecules early after transfection. To find the optimal time point in HEK293T cells, we edited 14 control variants (in 14 different genes) which are not expected to have an effect on expression and harvested at three time points post-transfection: 4 days, 6 days, and 9 days. At 4 days, the edited allele is depleted in the mRNA (Additional file 4: Figure S1a). However, this effect is lessened after 6 days and gone by 9 days. Therefore, we used the 9-day time point for the assay in order to analyze only the mRNA which has been transcribed post-editing.

Next, we assessed how technical variation in editing efficiency or PCR amplification may affect the robustness of the assay, using data from HEK293T cells. We analyzed how the homology-directed repair (HDR) rate affects the standard deviation of variant effect size between editing replicates of 49 variants (2 replicates for 23 and 3 replicates for 26 variants). We found that very low HDR is associated with a higher standard deviation between replicates (Fig. 1c). Therefore, in subsequent analyses, we discarded any variant with an HDR rate of less than 0.4% as determined by the frequency of the alternative allele in the gDNA. The HDR rate varies greatly between loci but is very well correlated between replicates of the same variant (Spearman’s rho = 0.96, *p* = 2.7 × 10^−14^, Additional file 4: Figure S1b), suggesting that the results of the assay are not strongly influenced by PCR amplification bias or variation in transfection efficiency. The HDR rate was not significantly correlated to NHEJ rate (Spearman’s rho = 0.15, *p* = 0.27) as has been observed before [45], and neither HDR nor NHEJ rate was significantly correlated to two published predictors of gRNA efficiency [39, 46] (Additional file 4: Figure S2). There was a minimal correlation between the gene expression level and the standard deviation of the effect size between replicates, which was reduced with the HDR filter of 0.4% (Additional file 4: Figure S1d). Altogether, the effect sizes of the two replicates are well correlated (Spearman’s rho = 0.60, *p* = 1.3 × 10^−3^, Fig. 1d). These results indicate that the polyclonal allelic expression assay can be used to robustly detect the regulatory effects of genetic variants even at relatively low HDR rates and in lowly expressed genes.

In order to determine the optimal set of negative control variants, we compared the distribution of effect sizes of the synthetic control variants (new variants created in the same genes as the stop-gained variants) and non-eQTL variants (common synonymous variants where eQTL effects were tested in GTEx and not observed) in HEK293T cells. The synthetic control variants have several outlier variants with large effect sizes that are consistent in replicates (Additional file 4: Figure S1c). This suggests that a subset of synthetic variants affect transcript levels and are thus not ideal for negative controls. The non-eQTL control variants, however, have effect sizes consistently close to zero (median aFC = − 0.009), demonstrating the utility of population data in selecting non-functional negative control variants. The variance of the synthetic controls was significantly greater than the variance of the non-eQTL controls (1.1 versus 0.038; *F* test *p* = 2.4 × 10^−8^). The non-eQTL variants were thus utilized as the control distribution for comparison with the stop-gained and eQTL variants tested with the assay.

**Fig. 2:**
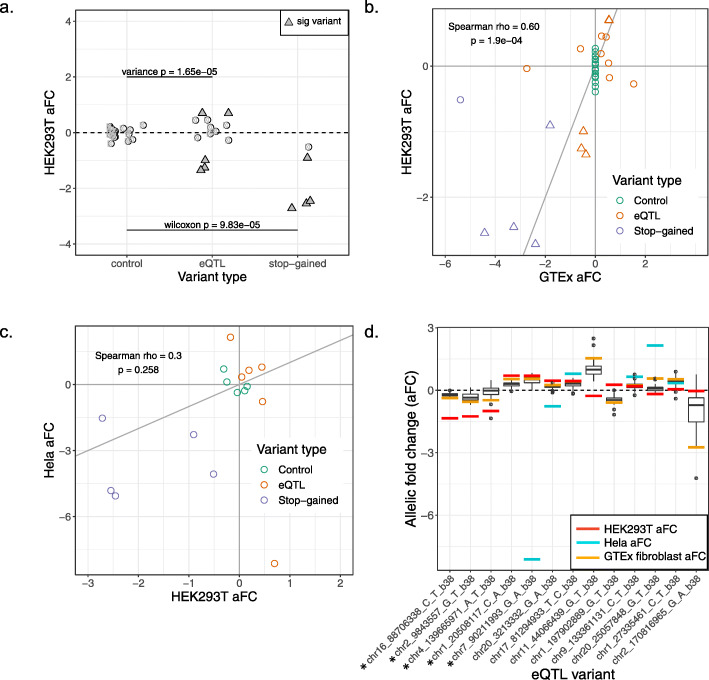
Stop-gained and eQTL variants from GTEx show allele-specific regulatory effects on expression. **a** Effect size of non-eQTL control, eQTL, and stop-gained variants after editing with the polyclonal allelic expression assay. Triangular points mark variants whose effect sizes significantly deviate from the control distribution. **b** Correlation between the effect sizes of variants in GTEx and effect sizes from the polyclonal allelic expression assay. **c** Correlation between the effect sizes of variants in HEK293T cells and in HeLa cells from the polyclonal allelic expression assay. **d** eQTL effect size (aFC) in GTEx tissues for the 13 edited eQTL variants shown as boxplots, with lines indicating the median effect size in GTEx fibroblasts and in the assay. Asterisks mark variants which were significant in the assay in HEK293T cells

In order to analyze the effects of genetic variants on gene expression levels using the polyclonal allelic expression assay, we first analyzed the rare stop-gained variants from GTEx that are expected to trigger NMD. As a group, the stop-gained variants show the expected negative effect sizes as compared to the control distribution (Wilcoxon *p* = 9.8 × 10^−5^, Fig. 2a). Four of these variants individually deviate significantly from the control distribution (Bonferroni-corrected *z* test *p* < 0.05, Fig. 2a and Additional file 3: Table S3). These results demonstrate our ability to capture NMD effects with the assay.

Next, we extended the assay to assess putatively causal eQTL variants within transcripts using GTEx fibroblast eQTLs, having established that transcript annotations are enriched in these eQTLs (Additional file 4: Figure S3). We chose fibroblasts because GTEx fibroblast transcriptome expression is highly correlated with that of HEK293 cells (rho = 0.68, *p* < 2.2 × 10^−16^, Additional file 4: Figure S4). The 33 eQTL variants chosen for editing are located within the transcript of the eGene with which they are associated and have a high posterior probability of causality based on CAVIAR fine mapping. After editing and QC filtering, 13 eQTL variants remained (Additional file 3: Table S3). The variance of the effect size of the eQTL variants was significantly higher than that of the control variants (0.49 versus 0.038; *F* test *p* = 1.7 × 10^−5^; Fig. 2a), which suggests that the edited eQTL variants as a whole have a greater regulatory effect than the edited control variants. Ten of the 13 variants have an effect in the same direction as the GTEx eQTL effect. Five of the eQTL variants are individually significantly different from the control distribution (Fig. 2a and Additional file 3: Table S3), and all five of these variants have an effect in the same direction as in GTEx. Additionally, there is a significant correlation between the effect size of the edited stop-gained, non-eQTL control, and eQTL variants and their effects in GTEx (Spearman’s rho = 0.60; *p* = 1.9 × 10^−4^, Fig. 2b), again indicating that the assay captures regulatory effects seen in the population.

In order to establish the robustness of the assay in a different cell line and analyze the potential cell type-specific effects, we replicated the assay for a total of 45 variants in HeLa cells, with 6 eQTL variants, 5 GTEx stop-gained variants, and 5 synonymous control variants passing QC (Additional file 3: Table S3). Due to the small number of control variants in this replication set, we analyzed variant effect sizes rather than distinguishing significant variants in HeLa cells. The effect sizes measured in HEK293T cells and HeLa cells were largely consistent (rho = 0.3, *p* = 0.258), with all stop variants showing the same direction of effect (Fig. 2c) and small effects of the control variants in HeLa cells. As in HEK293T cells, the HeLa effect sizes showed a correlation with GTEx effect sizes (Spearman’s rho = 0.42, *p* = 0.074; Additional file 4: Figure S5d), indicating that the assay is robust.

We expect that the lack of effect for some of the eQTL variants is due to the variants not actually being the causal regulatory variants of their association signals. Additionally, our cell line may not perfectly recapitulate the genetic regulatory effects of GTEx fibroblast samples. To investigate this, we looked at the variation in the effect size between GTEx tissues for each of the eQTL variants (Fig. 2d), as well as the variation of the effect sizes between HEK293 and HeLa cells (Fig. 2c, Additional file 4: Figure S5d). We also looked at inter-individual variation within fibroblast samples in GTEx, which may reflect more subtle cell type-specific genetic effects as well as the effects of other regulatory variants that the individuals may have. We measured the effect size in eQTL heterozygotes based on the allelic imbalance within the gene body (Additional file 4: Figure S5c), with eleven of the eQTL variants having sufficient data for this analysis. For all five significant variants in HEK293T cells, there is an agreement in direction between the polyclonal HEK293T aFC, median heterozygous aFC, and eQTL aFC. Some of the variations between the effect sizes observed in HEK293T and HeLa cells may be attributable not only to noise but also cell type-specific regulatory effects. Several of the other variants demonstrate a large range of effects in GTEx both across tissues and across individuals (Fig. 2d and Additional file 4: Figure S5c). The observed effect in the cell line, like an individual or tissue, is likely to fall somewhere in a spectrum of possible effects.

**Fig. 3:**
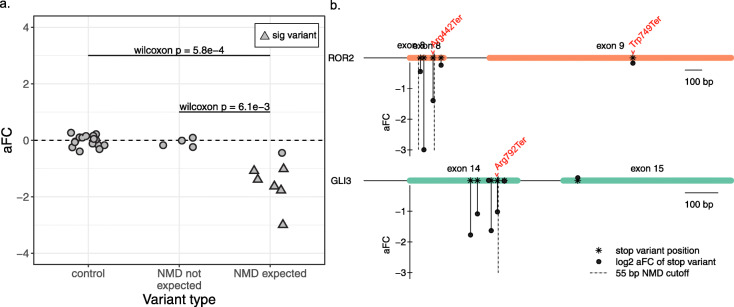
The polyclonal assay effectively detects NMD in disease-associated genes. **a** Effect size in control variants, stop-gained variants after the NMD threshold, and stop-gained variants before the NMD threshold. Triangular points mark variants whose effect size significantly deviates from the control distribution. **b** The last two exons of NMD disease genes *ROR2* and *GLI3*, showing the effect size (*y*-axis) and position in the transcript (*x*-axis) for each successfully edited variant. Disease-associated variants from ClinVar are labeled in red

In order to apply our assay to the detection of nonsense-mediated decay triggered by disease-associated variants, we introduced stop-gained variants into two disease-associated genes: *ROR2* and *GLI3*, primarily in HEK293T cells. Seven of the edited stop-gained variants are before the 55-bp threshold and were therefore expected to trigger NMD. Of these variants, all seven resulted in negative effect sizes, and the distribution of these variants was significantly different from that of both the four variants which were not expected to trigger NMD (Wilcoxon *p* = 6.1 × 10^−3^) and the non-eQTL control variants (Wilcoxon *p* = 5.8 × 10^−4^, Fig. 3a). When tested individually, six of the seven expected NMD variants are significantly different from the control distribution, indicating that we can sensitively detect NMD and NMD escape across the 55-bp boundary in these two genes (Fig. 3a and Additional file 3: Table S3).

In addition to the newly created stop-gained variants, we also included disease-causing stop-gained variants from ClinVar. The Arg442Ter mutation in *ROR2* results in a stop codon right before the predicted NMD cutoff and is associated with the recessively inherited Robinow syndrome. We observe a significant negative effect of this variant (aFC = − 1.39, Bonferroni-corrected *z* test *p* = 2.2 × 10^−11^), which is consistent with NMD and the clinical manifestation of the disease (Fig. 3b). In contrast, the variant Trp749Ter is associated with dominant type B brachydactyly and falls after the NMD cutoff in the transcript. Our assay shows that Trp749Ter does not affect the expression level of *ROR2* and therefore does not appear to be triggering NMD (aFC = − 0.17, corrected *p* = 1). The one disease variant tested in *GLI3*, Arg792Ter, falling immediately before the predicted border of NMD escape, shows evidence of triggering NMD with a negative effect size in the assay (aFC = − 1.02, corrected *p* = 3.6 × 10^−6^). This result is consistent with the clinical features of this variant, with Grieg cephalopolysyndactyly syndrome thought to be caused by haploinsufficiency in the gene *GLI3*. The results of editing stop-gained variants in these disease genes indicate that there is a sharp cutoff of NMD/NMD escape at the previously described 50–55-bp threshold and pinpoint the immediate molecular mechanism of NMD/NMD escape for these disease variants. For the three variants that were also tested in HeLa cells and passed QC, the NMD signal was consistent with that in HEK293T cells (Additional file 4: Figure S5d). Additionally, the results demonstrate the potential for utilizing this assay to assess whether a variant of clinical interest triggers NMD when it falls close to the threshold of NMD escape.

## Discussion

In this study, we described a method utilizing CRISPR/Cas9 genome editing and targeted sequencing to validate regulatory variants without the need for isolating monoclonal cell lines. We demonstrated our ability to reliably detect the effects of stop-gained variants in the general population and in disease cases with the assay. The ability to experimentally assess the effect of potentially disease-causing stop-gained variants could lead to not only better understanding of the rules of NMD/NMD escape, but also more accurate diagnosis and prognosis. The American College of Medical Genomics recommends caution interpreting the pathogenicity of stop-gained or frameshift variants of unknown significance, especially in cases where the variant is in an exon which might be alternatively spliced, or close to the 3′ end of the transcript [47]. Even though RNA analysis from patients is increasingly used to support variant interpretation [27, 48, 49], establishing causality has been difficult since the lower expression of a mutant haplotype or gene could be driven by other genetic or environmental factors. Our approach can provide evidence that the introduction of the specific variant in question underlies transcript-level changes, thus reducing the ambiguity of variant effects. Furthermore, for genes where NMD/NMD escape is clinically relevant, saturation editing at the 50–55-bp border could build a high-resolution reference for variant interpretation, as it is currently largely unknown how sharp and variable this border is across genes.

This polyclonal assay has the ideal throughput for identifying causal variants from a list of a few to several dozen candidate variants discovered from a rare genetic study. It would be feasible to perform the polyclonal assay on a number of potential regulatory variants, sequencing mRNA and gDNA from the polyclonal culture, and then sort monoclonal cell lines from the same polyclonal culture for only the variants which demonstrate allele-specific regulatory activity. In this approach, the polyclonal assay narrows down the pool of variants to a reasonable number for in-depth follow-up with functional assays, protein quantification, etc. The straightforward nature of the assay makes it easily adoptable in any lab with tissue culture facilities and access to a sequencing instrument.

When we applied the polyclonal assay to eQTL variants, we detected increased variant effects on the expression levels as compared to controls, often in the same direction as the GTEx eQTL effect. Five of 13 variants had significant effects in HEK293T cells, all consistent with the GTEx eQTL data. This clearly demonstrates the ability of our assay to capture common regulatory variant effects. Some of the non-significant eQTL variants appear to have edited effect sizes consistent with GTEx in HEK293T and/or HeLa cells, but we lack the sensitivity to detect these small effects with confidence. In addition, some of the inconsistencies between the assay results and eQTL data are likely to originate from the eQTL data. Since we do not expect fine-mapping to always succeed in identifying the true causal variants at these loci, the undetected effects could represent these situations. Furthermore, with multiple eQTLs for the same gene being common [4], it is possible that eQTL effect sizes observed in populations reflect multiple regulatory variants in partial LD. Therefore, editing a single variant may not yield the same results as the full haplotype. When we looked at the aFC in heterozygous individuals for these variants in GTEx, we found a broad range of effect sizes, suggesting the presence of effects from multiple variants and potential modifiers that may not be captured by editing a single variant. Finally, assessing genetic regulatory effects even in closely matched cell lines does not necessarily capture effects measured in tissue samples. While this is likely to contribute to some of the differences, *cis*-eQTLs, especially in the transcribed region, are often highly robust across different tissues [4] and are expected to replicate in cell lines as well. We highlight that our approach maintains the genomic context of variants and native gene regulation. Thus, it does not suffer from the limitations of massively parallel approaches where discrepancies between eQTL and experimental data may be due to measuring genetic regulatory effects in artificial constructs [10, 11]. Altogether, more experimentation and further comparison of population and experimental results are required to fully understand the differences between experimental and population data.

Finally, we note that our assay is somewhat limited by HDR efficiency, which varies greatly between loci and led to filtering out a substantial number of targeted loci from our final analysis. Capturing the specific effect of the edited variant requires discarding any reads in the gDNA or cDNA which contain indels created through non-homologous end joining (NHEJ). Since NHEJ often dominates HDR in efficiency, this can result in low numbers of HDR reads. Research in improving the HDR rates in editing is ongoing [50–52], and likely HDR efficiency will be greatly improved in the future. Additionally, future improvements on base editor technology, which avoids the introduction of double-stranded breaks and therefore minimizes the risk of indels [53, 54], could also benefit this system and increase the sensitivity of the assay. Finally, while the assay can be applied to multiple cell lines to add robustness of the results and indicate cell type-specific variant effects, cell lines may not fully capture variant effects in vivo.

## Conclusions

In summary, we have presented a method to validate the allele-specific effects of regulatory variants using CRISPR/Cas9 in human cell lines. When applied to eQTL variants, we see an increased regulatory effect over the control variants, suggesting we are capturing the allele-specific regulatory effects of these variants. Additionally, all of the significant eQTL variants have effects in the same direction as observed in GTEx, demonstrating the assay’s reliability in detecting eQTL effects. The assay is particularly robust in capturing variants triggering NMD across the genome and in rare disease genes, with potential applications in testing the effects of variants of unknown significance from rare variant studies.

## Supplementary information

**Additional file 1. Table S1.** Information on the variants chosen for editing.

**Additional file 2. Table S2.** Reference and alternate allele counts in cDNA and gDNA sequencing samples after editing variants.

**Additional file 3. Table S3.** Effect sizes of edited variants which passed QC filtering steps.

**Additional file 4.** Supplementary figure file containing Figure S1-S5.

## Data Availability

The sequencing dataset supporting the conclusions of the article is available in the figshare repository at 10.6084/m9.figshare.9883232 [55]. The laboratory protocol is available at 10.17504/protocols.io.7c6hize.
