# Supplementary material for: A polyclonal allelic expression assay for detecting regulatory effects of transcript variants
Source: Genome Med. 2020 Sep 11;12:79. doi: 10.1186/s13073-020-00777-8 (PMC7488413; doi:10.1186/s13073-020-00777-8)
Supplement: Supplementary file 4 — Additional file 4. Supplementary figure file containing Figure S1-S5. [file 13073_2020_777_MOESM4_ESM.pdf]

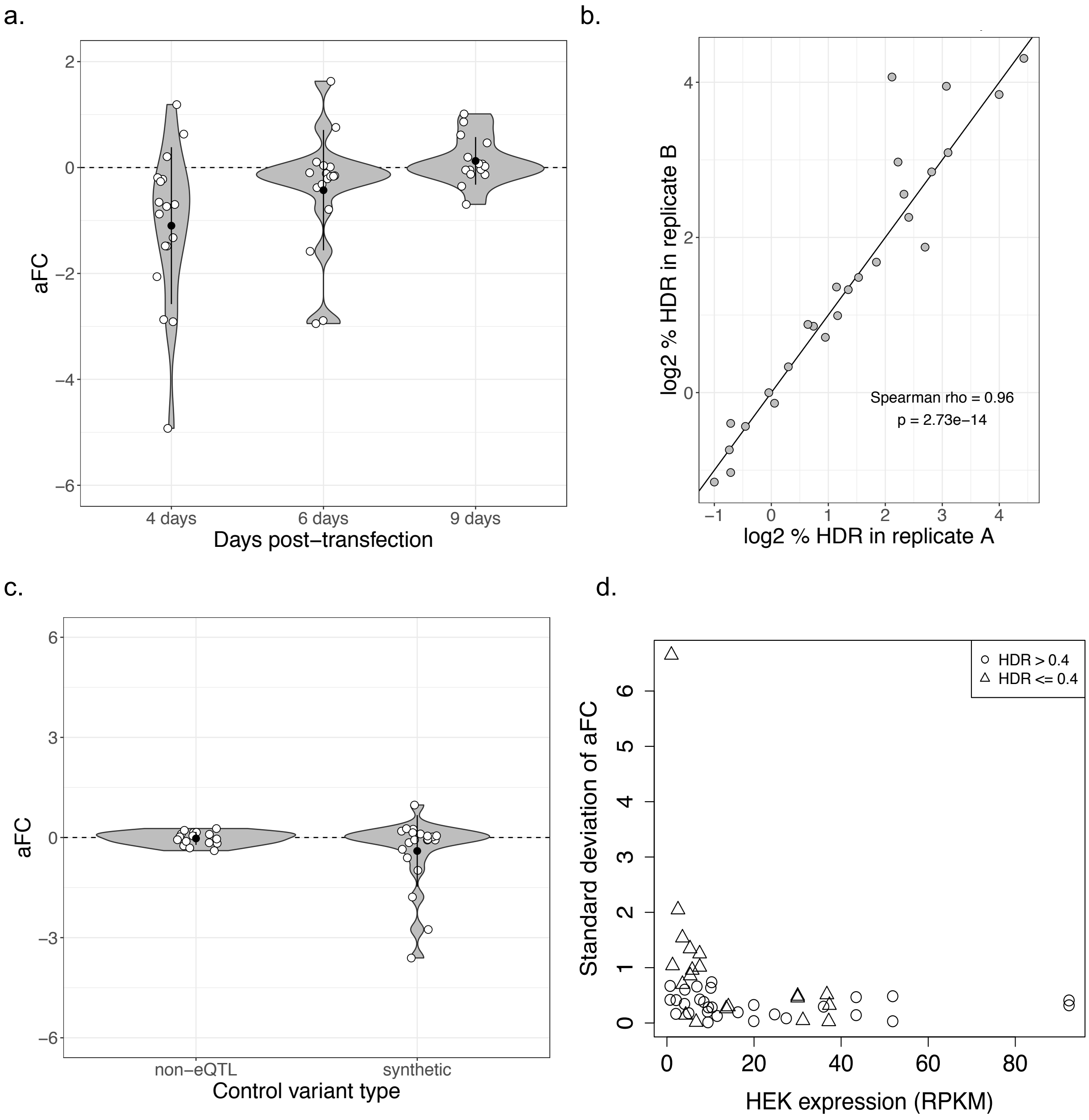


**Figure S1. Technical aspects of polyclonal allelic expression assay.** (a) Distribution of effect size for control variants over three timepoints post-transfection. (b) Homology direction repair rate in two replicates of control and experimental variants nine days after editing. (c) Distribution of effect size for different control variant types: synthetic control variants and GTEx synonymous non-eQTL control variants. (d) HEK expression versus standard deviation in effect size (aFC) for GTEx variants. With all variants, spearman rho = -0.435, p = 0.0018. After filtering for HDR, spearman rho = -0.29, p = 0.118.

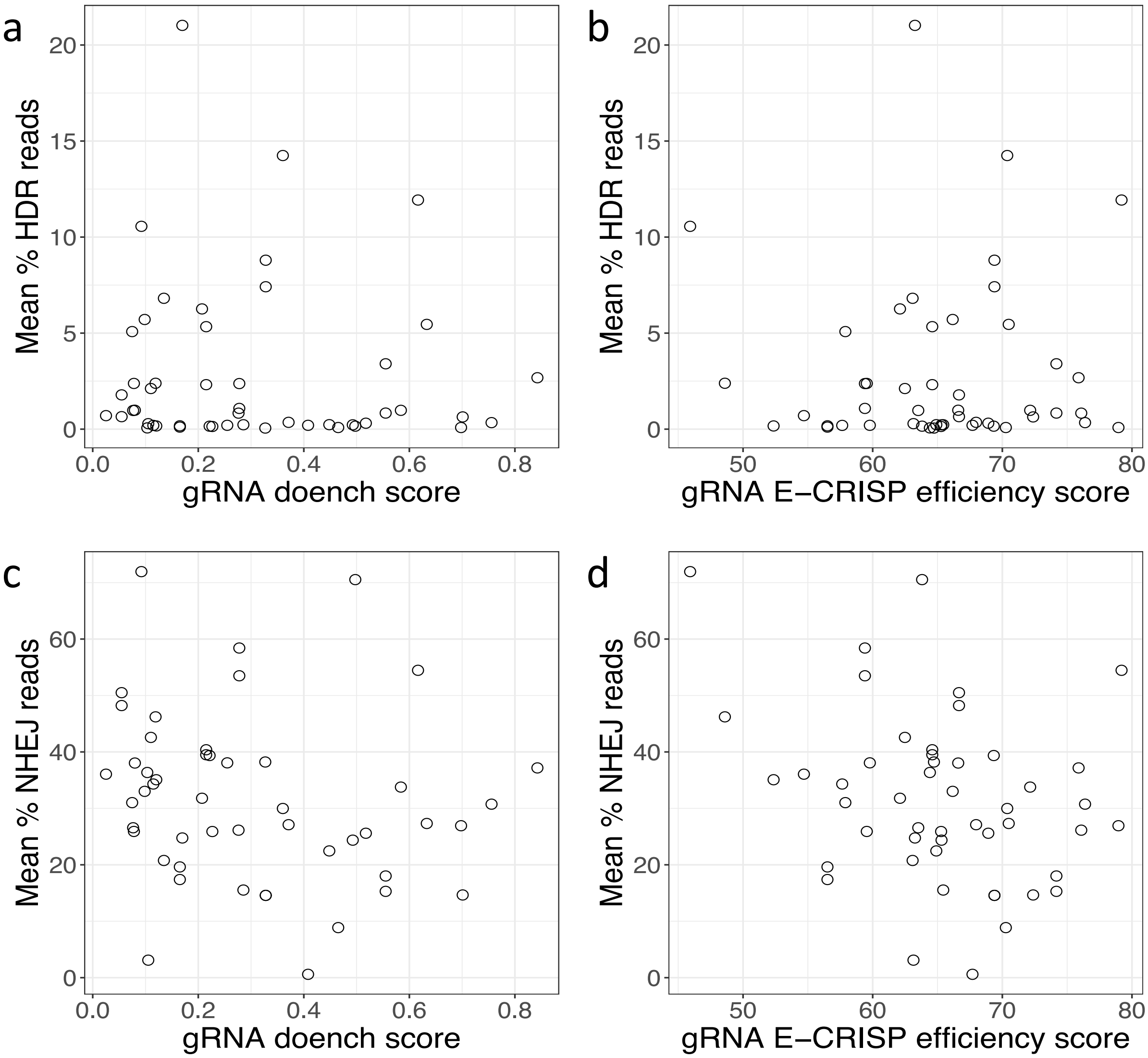


**Figure S2. Predicted versus observed editing efficiency in CRISPR edited variants. (**a) Predictive scores of gRNA editing efficiency based on Doench et al, 2016 versus observed homology-directed repair (HDR) rate (Spearman rho = -0.087, p = 0.55). (b) E-CRISP gRNA editing efficiency scores versus observed HDR rate (Spearman rho = 0.054, p = 0.71). (c) Doench et al gRNA editing efficiency scores versus non-homologous end joining (NHEJ) rate (Spearman rho = -0.27,p = 0.058). (d) E-CRISP gRNA editing efficiency scores versus NHEJ rate (Spearman rho = -0.27, p = 0.061).

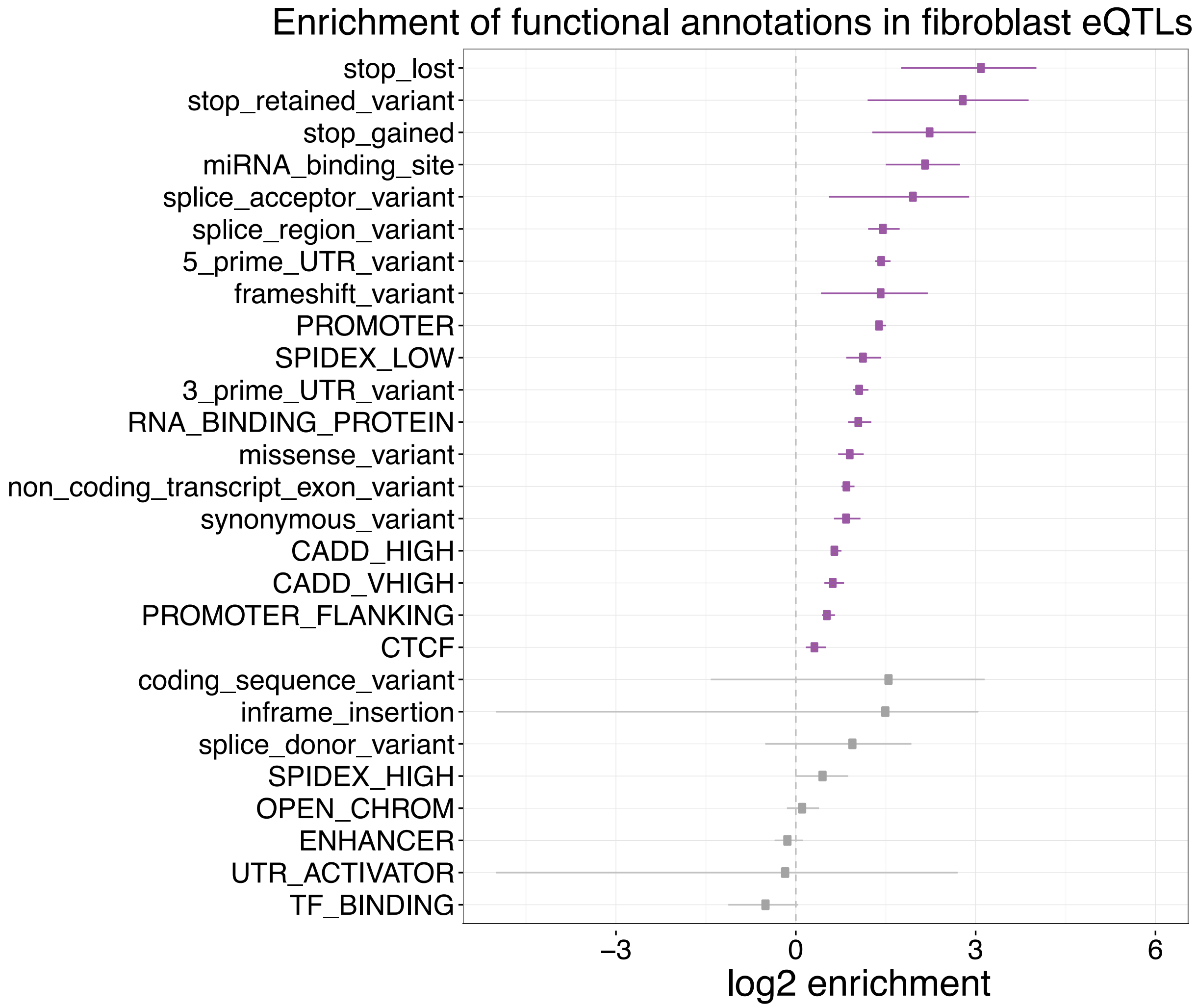


**Figure S3. eQTL variants demonstrate enrichment for annotations within the transcript.** fgwas enrichment of functional annotations in GTEx fibroblast eQTL variants. Significant annotations are colored in purple.

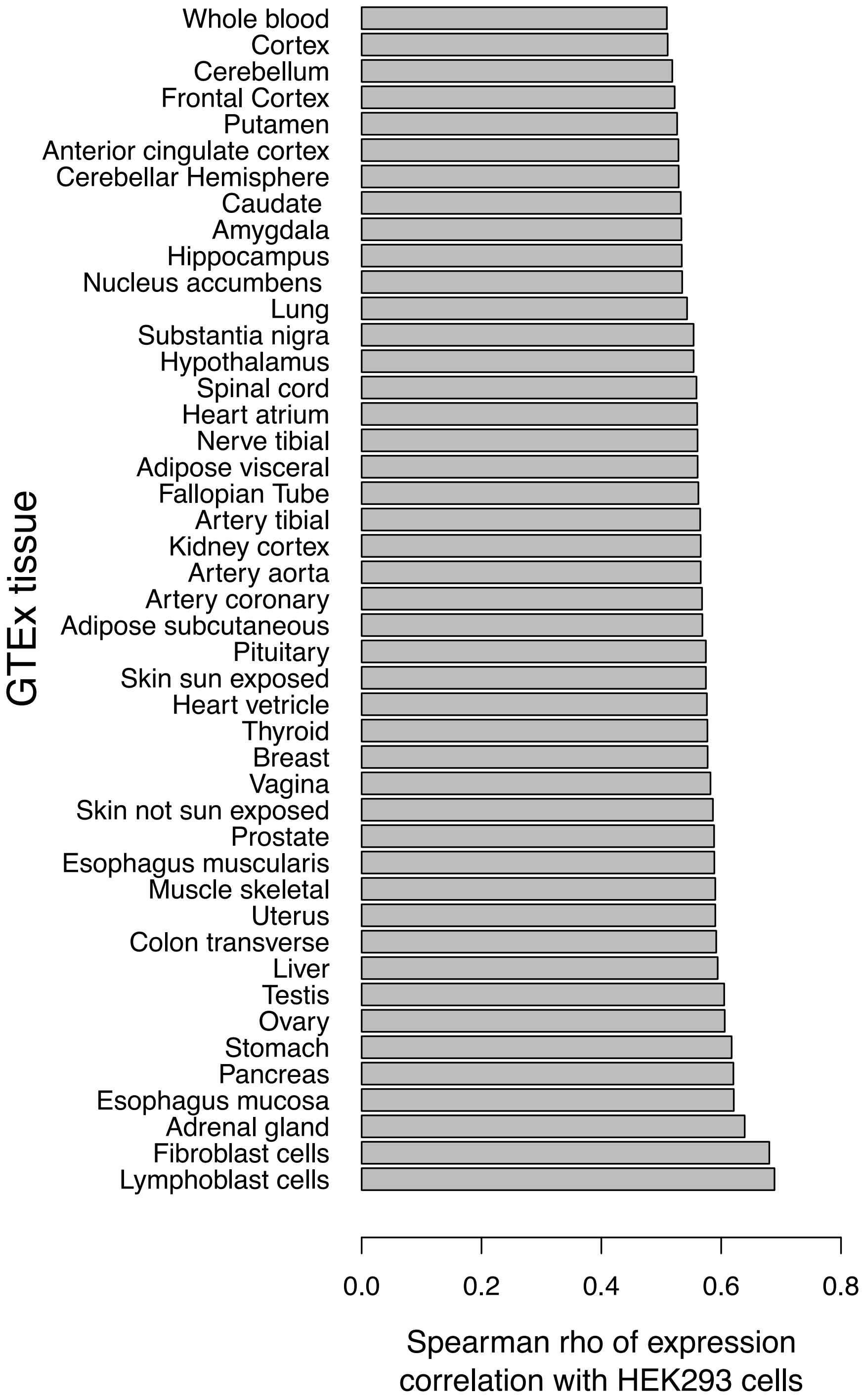


**Figure S4. Correlation of transcriptome expression between GTEx tissues and HEK 293 cells.**

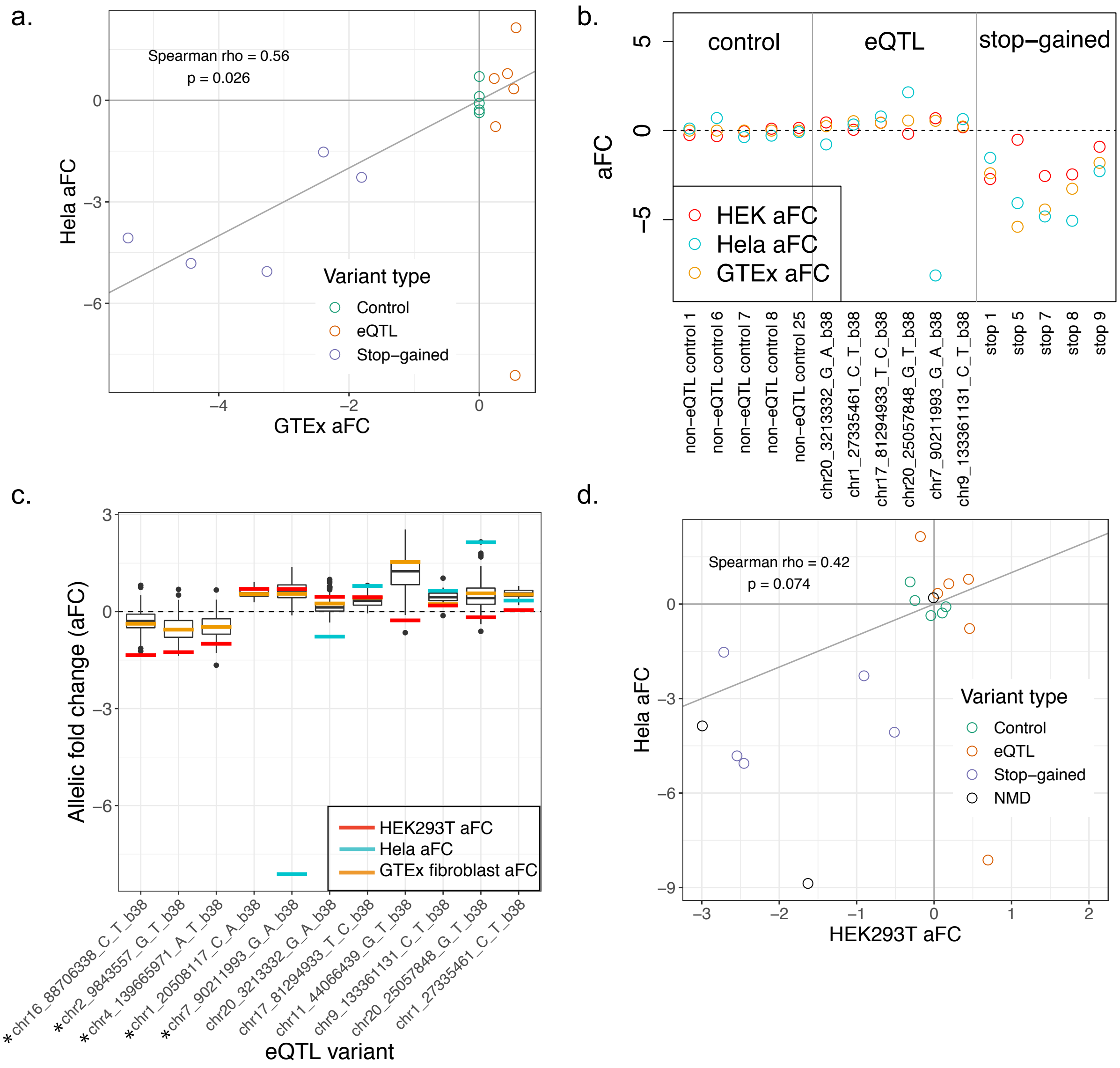


**Figure S5. Effects of variants in GTEx, HEK293T and HeLa cells.** (a) Effect size (aFC) of variants in HeLa cells versus in GTEx. (b) aFC in GTEx, HEK293T and HeLa cells for GTEx variants tested in both cell types. (c) aFC in GTEx fibroblasts, measured in eQTL heterozygous individuals for 11 of the edited eQTL variants shown as boxplots, with lines indicating the median effect size in GTEx fibroblasts, HEK293T and HeLa cells with the assay. Asterisks mark variants which were significant in HEK293T cells (d) aFC of all variants in HeLa cells versus in HEK293T cells, including three variants in the two Mendelian disease genes (“NMD”).
